# The Impact of Perceived Etiology, Treatment Type, and Wording of Treatment Information on the Assessment of Gastritis Treatments

**DOI:** 10.3389/fpubh.2020.00035

**Published:** 2020-02-25

**Authors:** Joachim Kimmerle, Aline Anikin, Martina Bientzle

**Affiliations:** ^1^Leibniz-Institut fuer Wissensmedien/Knowledge Media Research Center, Tübingen, Germany; ^2^Department of Psychology, University of Tuebingen, Tübingen, Germany

**Keywords:** health communication, health perceptions, treatment assessment, gastritis, experiment

## Abstract

How patients assess the suitability of a certain therapy for treating a disease depends on a variety of influencing factors. Three key factors are people's subjective perceptions of a disease, the type of treatment, and the kind of communication used to convey information. The study presented here was a randomized controlled experiment in which we examined these three factors. We used a mixed design where we manipulated perceived etiology of gastritis (biopsychosocial vs. biomedical) as a between-group factor, and treatment type (behavioral vs. pharmacological) and wording of treatment information (holistic vs. scientific) as within-group factors. We found that gastritis treatments that matched the perceived etiology of the illness were assessed to be more effective. Moreover, treatments that matched the perceived etiology enhanced participants' intention to undergo the treatment themselves and their willingness to recommend it to a person close to them. Finally, participants' intention to undergo the treatment was also enhanced when the wording of the treatment information matched the perceived etiology. We discuss the implications of our findings in terms of health communication and patient education.

## Introduction

People have to assess health-related information on a regular basis. This applies to information on the causes of health problems, the adequacy of treatment options, or the specific framing of health information. Previous research has shown that people's evaluation of treatment options depends on how they perceive the causes of particular health problems. In general, a treatment is more easily accepted, if people regard it to be directly related to what they consider the cause of the disease (1, 2). For example, people may have particular beliefs and assumptions about the etiology of a disease that can either be biopsychosocial or biomedical (3–6). *Biopsychosocial* considerations are particularly related to behavioral aspects, take patients' environment into account, and consider individuals from a holistic perspective. That is, the cause of a disease is considered to reflect life circumstances (5, 7, 8). Biopsychosocial causes would therefore include issues, such as psychological stress or unfavorable nutrition. From this etiological perspective, treatments should aim at solving health problems by addressing behavior options, such as stress reduction or a dietary change. *Biomedical* causes, in contrast, would include genetic components or viral and bacterial diseases. Pharmacological or surgical approaches would be considered relevant treatment options when the causes of a disease are considered to be biomedical.

Since previous research has also found that the particular wording of health-related information has an impact on how people deal with that information (9, 10), it is plausible that people are also sensitive to the match or mismatch of etiology and the wording of treatment information. Therefore, in the study presented here, we aimed to examine the interplay of people's perceived etiology with treatment type as well as the interplay of perceived etiology with the wording of treatment information. We used gastritis as an example, since this is an illness that has both biopsychosocial and biomedical causes (11, 12), and its treatment can be *behavioral* as well as *pharmacological* (13).

### Perceived Etiology and Type of Treatment

People's beliefs about an illness and its causes depend on the particular disease in focus (1). Regarding obesity, for instance, patients assume that there are hormonal causes, or that a slow metabolism is the cause (14). For depression, in contrast, patients report stress or their own personality as causes (15). Accordingly, those types of treatments that are in line with what is considered the cause are preferred (14, 16). If people consider their own lifestyle to be responsible for their obesity, they accept physical activity as a suitable intervention. In contrast, if they assume genetic reasons, they are less willing to change their diet or start exercising (2). In addition, people's motivation to follow a treatment is stronger if the treatment fits their own point of view (17). Patients who suffered from a myocardial infarction and attributed their heart attack to bad habits reported more lifestyle and nutrition changes after 6 months (18). What is particularly relevant here is that such causal attributions are not only modifiable (19) but also susceptible to experimental manipulation: Ogden and Jubb (1) used vignettes that either emphasized psychological or biomedical causes of several diseases. They found that treatments that were congruent to the causes were considered to be more effective.

The relationship between perceived etiology and the acceptance of a treatment is particularly relevant in the field of doctor-patient communication. In general, patients and their relatives prefer a patient-centered approach (20, 21). For the subject discussed here, this implies that doctors should not only provide information but should also take the patients' perspectives and assumptions into account (22, 23). Patient-centered communication would then yield several advantages, such as higher satisfaction, more frequent completion of treatments, and better outcomes (21, 24). Therefore, doctors should be aware of their own and their patients' cognitive representations of diseases and treatments. It might be problematic if doctors' assumptions differ from those of their patients. While people concerned tend to consider obesity to be a hormonal problem, physicians attribute it to wrong nutrition (14, 16). When such assumptions diverge, a physician might offer a treatment that would result in low treatment satisfaction, low compliance, or premature termination of a therapy.

On the basis of these considerations, we hypothesized that a match between people's perceived etiology of gastritis with the type of treatment would have a positive impact on their assessment of the treatment. This would be the case on several levels, that is, on their assessment of treatment *effectiveness*, their (hypothetical) intention to undergo the treatment themselves (*participation intention*), and their *recommendation* of this treatment to a person close to them. The match between perceived etiology and type of treatment would be applied to a case of a perceived biopsychosocial cause of gastritis combined with a behavioral treatment, and to a case of a perceived biomedical cause combined with a pharmacological treatment. Accordingly, we stated the following hypotheses:

Hypothesis 1a: When perceived etiology matches the type of treatment, people will assess the treatment to be more effective than with a mismatch.
Hypothesis 1b: When perceived etiology matches the type of treatment, people will be more willing to undergo the treatment themselves than with a mismatch.
Hypothesis 1c: When perceived etiology matches the type of treatment, people will be more willing to recommend this treatment to a person close to them than with a mismatch.

### Perceived Etiology and Wording of Treatment Information

When people deal with health information, it is not only the content of a health message that is important but also the particular wording of the message that is being communicated (25, 26). Tayler and Ogden (27), for example, found that when doctors used the term “heart failure” in a consultation, patients believed that this illness had more serious consequences, and they felt more anxious and depressed than when the condition was described using a euphemism. It was found in many studies that health information tailored to the perceptions and beliefs of patients achieved a greater effect than non-tailored information (for an overview see (4, 28, 29) showed that women perceived pro and con arguments about mammography screening to be more relevant when they were phrased in a way consistent with their own personal health concepts. Kimmerle et al. (9) found that women who were already inclined to participate in a mammography screening program recommended *scientifically worded* arguments to other women instead of *holistically worded* arguments.

Building on these previous findings, we expected that a match between people's perceived etiology of gastritis with the wording of treatment information would have a positive impact on their assessment of that treatment. A match of perceived etiology and wording of treatment information would be applied to a case of a perceived biopsychosocial cause of gastritis combined with a holistic wording, and to a case of a perceived biomedical cause combined with a scientific wording. Accordingly, we stated the following hypotheses:

Hypothesis 2a: When perceived etiology matches the wording of treatment information, people will assess the treatment to be more effective than with a mismatch.
Hypothesis 2b: When perceived etiology matches the wording of treatment information, people will be more willing to undergo the treatment themselves than with a mismatch.
Hypothesis 2c: When perceived etiology matches the wording of treatment information, people will be more willing to recommend this treatment to a person close to them than with a mismatch.

## Methods

### Participants and Design

The study was carried out with 87 participants (Mean [M]_age_ = 25.61 years old, SD = 9.65 years old, age range: 19–66 years old) who were recruited via e-mail through a database of voluntary participants. This database contained almost exclusively university students. Sixty participants had a university-entrance diploma and 22 had a graduate degree. The research reported here was performed in accordance with the Declaration of Helsinki and had full approval by the ethics committee of the Leibniz-Institut fuer Wissensmedien (Approval No: LEK 2017/025). All of the participants provided written informed consent. We applied a 2 × 2 × 2 mixed design. We manipulated perceived etiology of gastritis (biopsychosocial vs. biomedical) as a between-group factor, and treatment type (behavioral vs. pharmacological) and wording of treatment information (holistic vs. scientific) as within-group factors. Forty four participants were randomly assigned to the biopsychosocial and 43 were assigned to the biomedical etiology of gastritis condition. Dependent variables were treatment effectiveness, participation intention, and treatment recommendation.

### Procedure and Material

Participants received an introduction text that informed them about issues of privacy protection and the general purpose of the study. Then we asked demographic questions about age and education. After this, the participants were randomly assigned to one of two experimental conditions in order to manipulate their perceived etiology of gastritis: They received either a patient vignette about a patient who suffered from gastritis due to biopsychosocial causes or a vignette about a patient who suffered from gastritis due to biomedical causes (see below for details). Following this experimental manipulation, we presented four manipulation check items, where participants had to indicate whether they perceived the patient's illness to have biopsychosocial or biomedical causes.

After this, the participants rated four potential treatments for gastritis (two behavioral and two pharmacological treatments) with regard to each of the three dependent variables (effectiveness, participation intention, and recommendation). We systematically permuted the sequence of the presentation of the treatments as well as the particular wording of the treatment information across participants: For each participant one behavioral treatment was worded holistically and the other behavioral treatment was worded scientifically; the same applied to the two pharmacological treatments (see below for details). Participation in this study took about 15–20 min.

#### Patient Vignettes

Both vignettes described the fictitious case of a patient who suffered from gastritis. In both cases the patient was introduced with the same symptoms:

“*Patient xy has been suffering from acute gastritis (inflammation of the lining of the stomach) for some time. This manifests itself through loss of appetite, burning pain in the stomach, and pressure in the stomach area.”*

The biopsychosocial patient vignette continued as follows:

“*The ailment started after a conflict with his boss. Since Mister xy spends the day in the office until late at night, he only survives this with a lot of coffee. At lunchtime he just eats something fast; in the evening he prepares a ready-made meal.”*

The biomedical patient vignette continued as follows:

“*His mother and his grandfather suffered from this ailment before him. All of them were diagnosed with an infection with the bacterium Heliobacter pylori. Moreover, Mister xy has been struggling with digestive problems and bile reflux for years.”*

#### Treatment Types

We aimed to find examples of both types of treatments (behavioral and pharmacological) that unbiased people would regard as equally suitable. Therefore, we pre-tested 21 different options for treating gastritis with 26 participants. From this pre-test we selected two behavioral and two pharmacological treatments that were rated as comparably suitable. The two behavioral treatments were “*avoiding spicy food”* and “*relaxation exercises.”* The pharmacological treatments were “*ingestion of acid blockers”* and “*ingestion of antibiotics.”*

#### Wording of Treatment Information

The information about the treatment methods was either worded holistically or scientifically. The holistic wording was: “*[Treatment] support(s) the healing process of gastritis and improve(s) the quality of life.”* The scientific wording was: “*Scientific studies have shown that [treatment] support(s) the healing process of gastritis.”*

### Measures

The manipulation check consisted of four items. Participants rated on 6-point Likert scales (1 = *I don't agree at all* to 6 = *I fully agree*) to what extent they considered the illness to have biomedical, genetical, behavioral, and mental causes.

For the measurement of the three dependent variables participants rated the respective items on 6-point Likert scales (1 = *I don't agree at all* to 6 = *I fully agree*). To measure participants' assessment of treatment *effectiveness*, we used three items: “This treatment makes sense as a therapy for gastritis,” “I think this treatment is effective as a therapy for gastritis,” “I think this treatment is convincing as a therapy for gastritis.” We measured participants' *participation intention* by asking them whether they would undergo this treatment if they suffered from gastritis. Finally, we captured data on whether participants would give a *recommendation* of this treatment to a close person.

### Analysis

We calculated a multivariate analysis of variance (MANOVA) for conducting the manipulation check. We also used mixed analyses of variance (ANOVAs) to examine the impact of perceived etiology, treatment type, and wording of treatment information on the three dependent variables regarding treatment assessment. The level of significance was set to α = 0.05.

## Results

### Manipulation Check

The biopsychosocial patient vignette and the biomedical patient vignette differed significantly in all of the four manipulation check items. In the biomedical condition, participants agreed to a greater extent that the illness had *biomedical causes* (*M* = 4.51, *SD* = 1.49) than in the biopsychosocial condition (*M* = 3.66, *SD* = 1.35), *F*_(1, 85)_ = 7.87, *p* = 0.006. In the biomedical condition, participants also agreed more strongly that the illness had *genetic causes* (*M* = 4.23, *SD* = 1.44) than in the biopsychosocial condition (*M* = 2.59, *SD* = 1.15), *F*_(1, 85)_ = 34.52, *p* < 0.001.

The reversed pattern was found for the behavioral and mental causes. In the biopsychosocial condition participants agreed to a greater extent that the illness had *behavioral causes* (*M* = 4.82, *SD* = 1.04) than in the biomedical condition (*M* = 2.95, *SD* = 1.48), *F*_(1, 85)_ = 46.42, *p* < 0.001. Finally, in the biopsychosocial condition participants also agreed more strongly that the illness had *mental causes* (*M* = 4.77, *SD* = 1.08) than in the biomedical condition (*M* = 2.81, *SD* = 1.42), *F*_(1, 85)_ = 52.83, *p* < 0.001.

### Perceived Etiology and Type of Treatment

**Figure 1:**
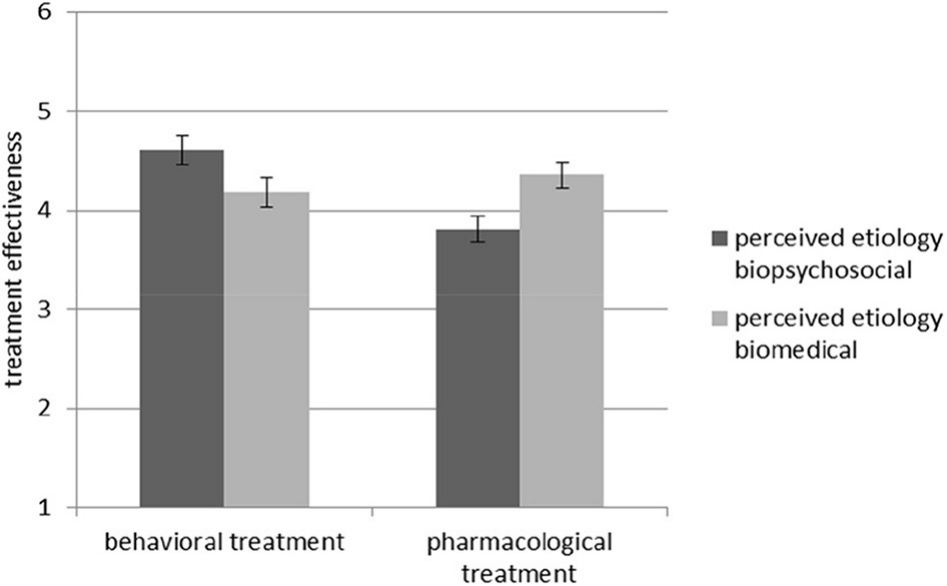
Interaction effect between perceived etiology and treatment type regarding *treatment effectiveness*. Standard errors are represented by the error bars attached to each column.

In Hypothesis 1a we had assumed that when perceived etiology matches the type of treatment, people will assess the treatment to be more effective than with a mismatch. The ANOVA with *treatment effectiveness* as the dependent variable supported this hypothesis, yielding an interaction effect of perceived etiology and treatment type, *F*_(1, 85)_ = 11.17, *p* = 0.001, _*part*._ η^2^ = 0.12 (see Figure 1).

**Figure 2:**
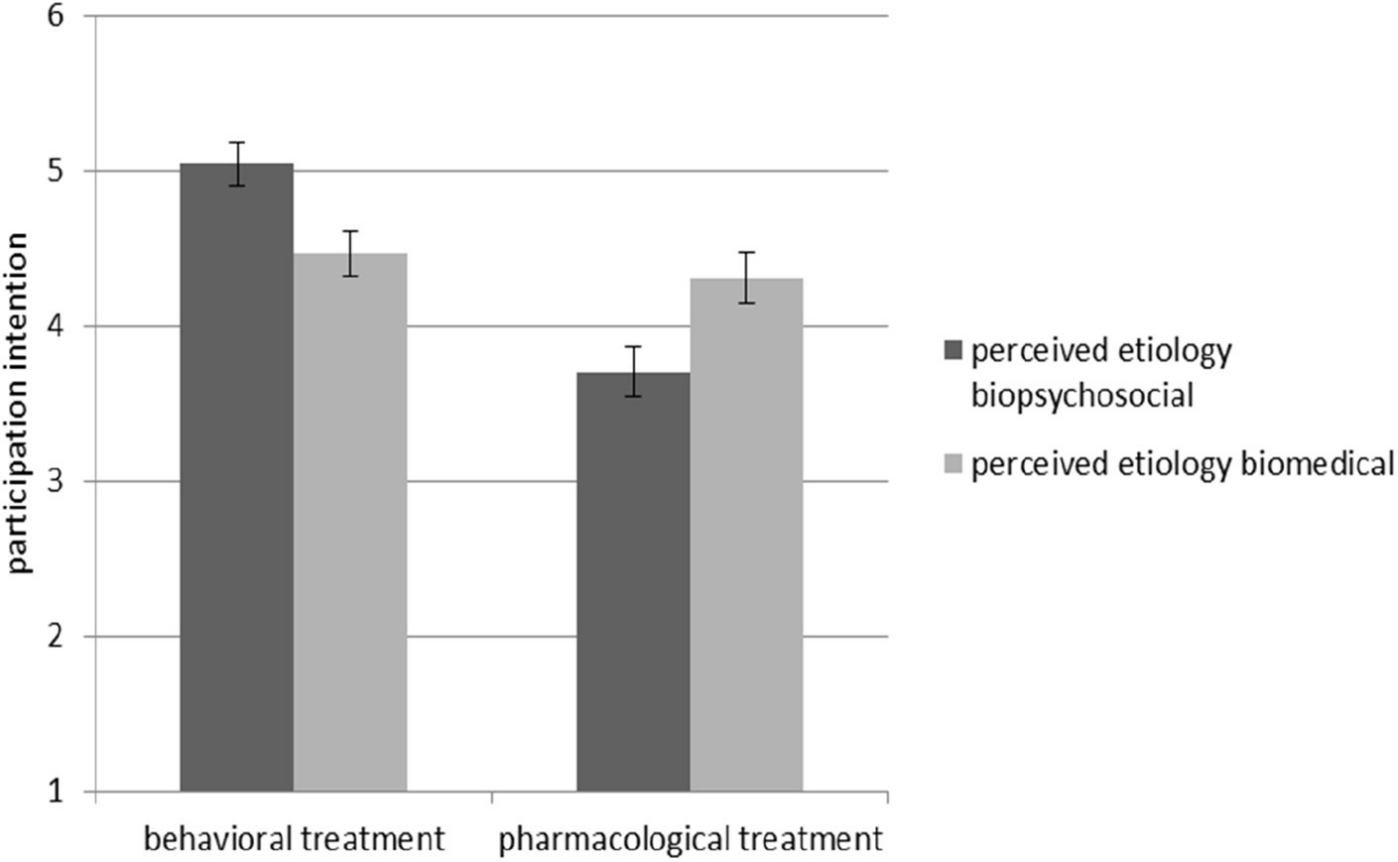
Interaction effect between perceived etiology and treatment type regarding *participation intention*. Standard errors are represented by the error bars attached to each column.

Hypothesis 1b stated that when perceived etiology space matches the type of treatment, people will be more willing to undergo the treatment themselves than with a mismatch. The ANOVA with *participation intention* as the dependent variable supported this hypothesis, indicating an interaction effect of perceived etiology and treatment type, *F*_(1, 84)_ = 14.67, *p* < 0.001, _*part*_ η^2^ = 0.15 (see Figure 2).

**Figure 3:**
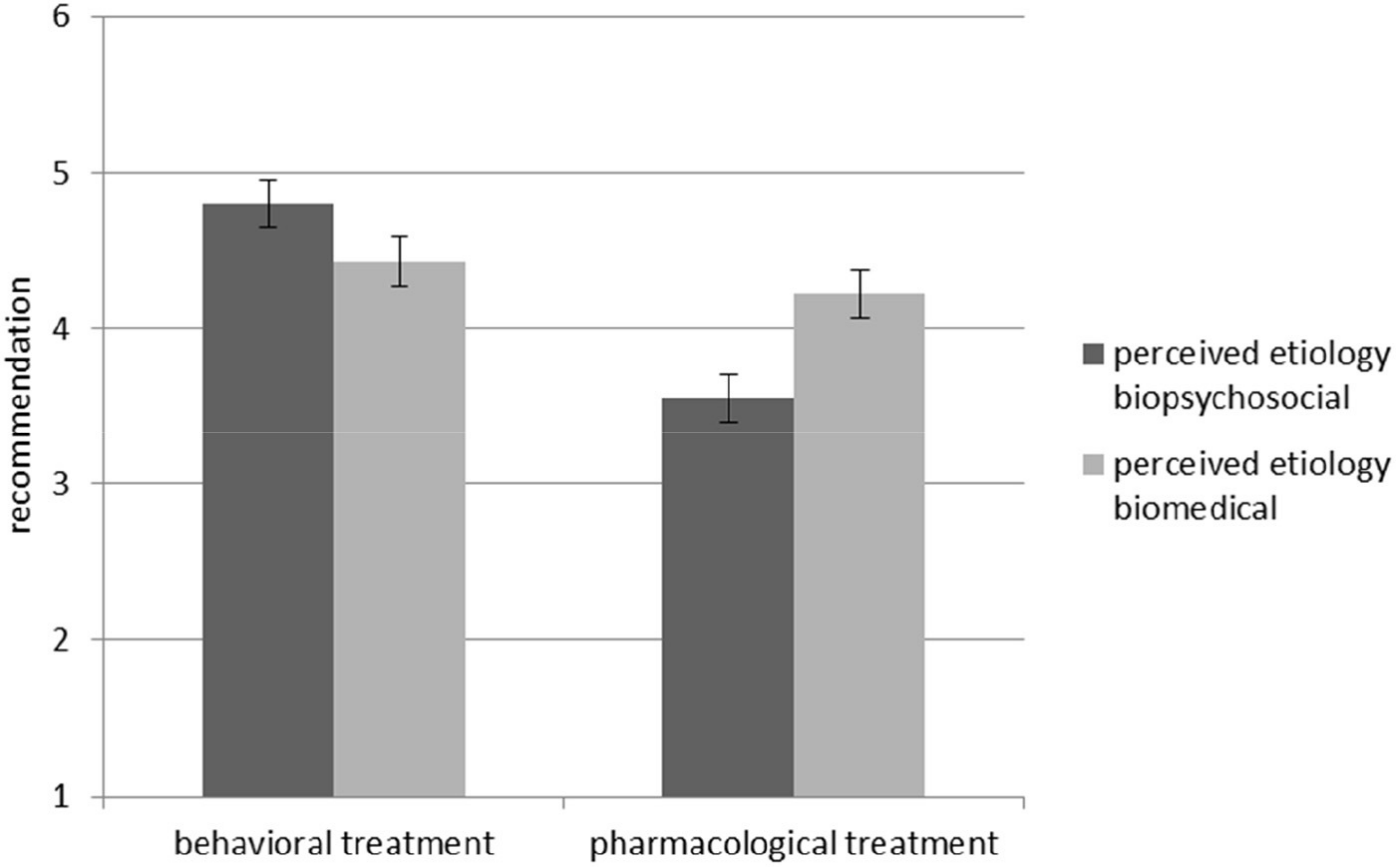
Interaction effect between perceived etiology and treatment type regarding *recommendation*. Standard errors are represented by the error bars attached to each column.

In Hypothesis 1c we had assumed that when perceived etiology matches the type of treatment, people will be more willing to recommend this treatment than with a mismatch. The ANOVA with *recommendation* as the dependent variable supported this hypothesis by showing an interaction effect of perceived etiology and treatment type, *F*_(1, 85)_ = 11.84, *p* = 0.001, _*part*_ η^2^ = 0.12 (see Figure 3).

### Perceived Etiology and Wording of Treatment Information

In Hypothesis 2a we had assumed that when perceived etiology matches the wording of treatment information, people will assess the treatment to be more effective. The ANOVA with *treatment effectiveness* as the dependent variable did not supported this hypothesis; there was no interaction effect of perceived etiology and wording of treatment information, *F*_(1, 85)_ = 1.26, *p* = 0.265.

**Figure 4:**
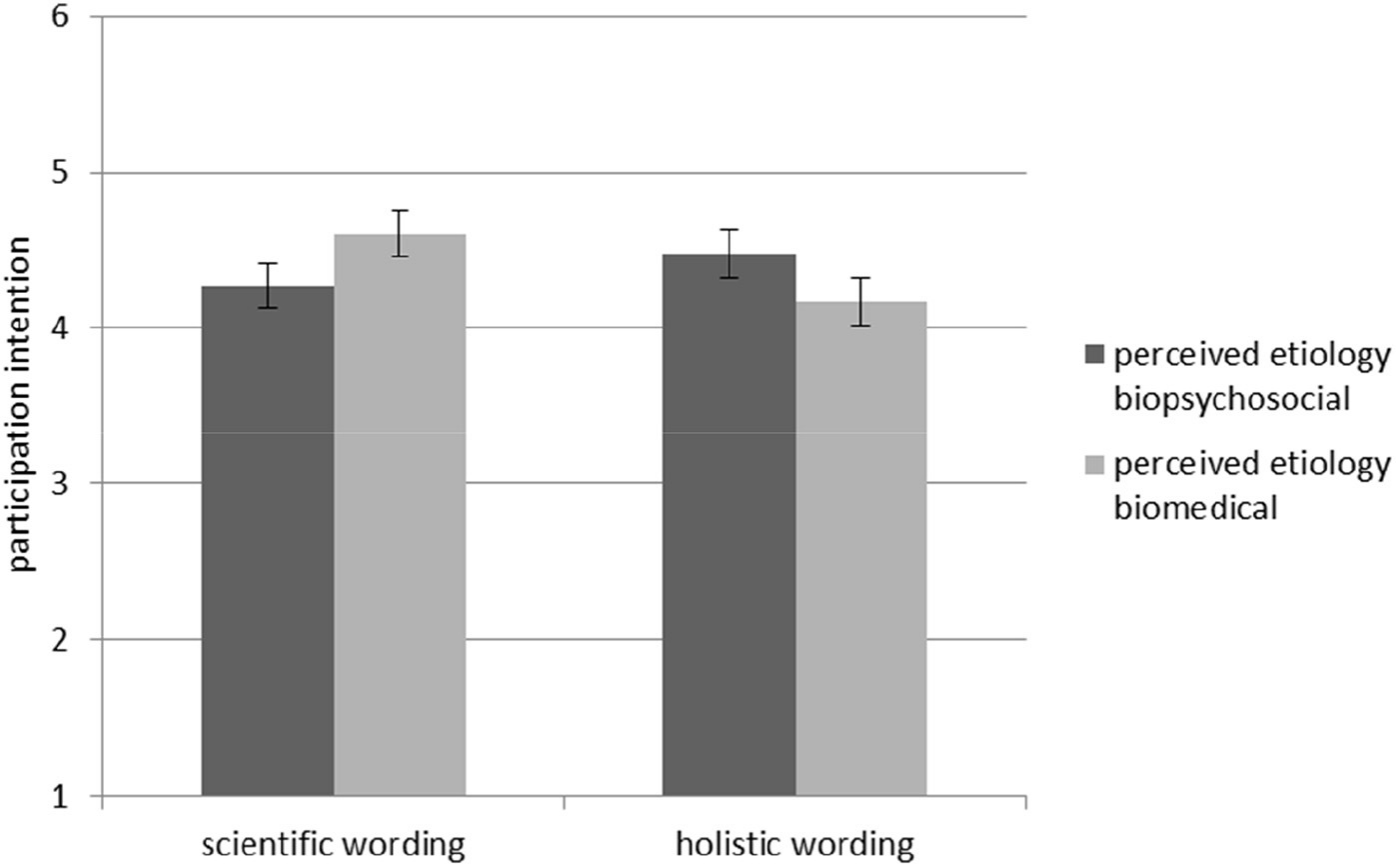
Interaction effect between perceived etiology and wording of treatment information regarding *participation intention*. Standard errors are represented by the error bars attached to each column.

Hypothesis 2b stated that when perceived etiology matches the wording of treatment information, people will be more willing to undergo the treatment themselves than with a mismatch. The ANOVA with *participation intention* as the dependent variable supported this hypothesis, indicating an interaction effect of perceived etiology and wording of information, *F*_(1, 84)_ = 4.63, *p* = 0.034, _*part*_. η^2^ = 0.05 (see Figure 4).

In Hypothesis 2c we had assumed that when perceived etiology matches the wording of treatment information, people will be more willing to recommend this treatment. However, the ANOVA with *recommendation* as the dependent variable found no interaction effect, *F*_(1, 85)_ = 2.24, *p* = 0.138.

## Discussion

The objective of this study was to examine the impact of the perceived causes of an illness, potential treatments, and the wording of the information about those treatments on how people assess the treatments in terms of effectiveness, how willing they are to undergo the treatments and how ready they are recommended to others. Our findings provide insight into the interplay between perceived etiology and potential treatments and the wording of information about treatments. The findings show that the evaluation of certain medical treatment methods and the consequences resulting from that evaluation, such as the willingness to take action accordingly, is influenced by a number of factors and their interaction. People make their decision about certain medical treatments depending greatly upon what they see as the actual cause of the disease and the context in which the information is embedded.

This has consequences for the design of information materials in health communication and for the way doctors educate their patients. For example, if patients assume behavioral causes for a disease, they are more willing to use behavioral methods to control the disease than if they assume genetic causes [cf. (2)]. Accordingly, physicians and health communicators should ensure that they have a correct understanding of the implicit or explicit health-related assumptions of their patients and people they need to communicate with [cf. (4)]. In this way they can reach their dialogue partners more optimally.

A limitation of this study is that treatments and causes of a disease were presented in isolation in order to illustrate the influence on the perceptions of the participants. In real-life settings, combinations of behavioral and pharmacological treatments are conceivable, especially in the case of gastritis. The avoidance of spicy food and an acid inhibitor could be applied in combination. Future studies should look at decision situations in which different treatments can be combined. Another limitation of the present study is that the participants themselves were not affected by gastritis. We cannot predict how people in an acute situation might make a decision about their disease. Future studies should also consider the influencing factors identified here in real patients. Finally, further studies should examine more closely the influence of perceived causes and the type of communication on the choice of treatments also in the context of other diseases. Above all, consideration should be given to investigating other diseases with well-chosen interventions to assess perceptions of treatment options, and with clearly formulated information about treatments to assess kinds of communication.

## Conclusion

In this study it could be shown that the representation of the causes of a disease has an influence on the choice of therapy. Treatments that are consistent with the assumed causes are preferred and evaluated as more effective. Therefore, it is very important for the application practice, for example in physician-patient discussions, that the treating physicians know the individual views of the patients. Doctors and other healthcare professionals need to consider these individual patient views and take them into account in the decision-making process in order to work with patients to find the best and most accepted course of treatment.

## Data Availability Statement

The datasets generated for this study are available on request to the corresponding author.

## Ethics Statement

The studies involving human participants were reviewed and approved by Ethics committee of the Leibniz-Institut fuer Wissensmedien. The patients/participants provided their written informed consent to participate in this study.

## Author Contributions

JK, AA, and MB made substantial contributions to the conception and design of the work, approved the final version to be published and agreed to be accountable for all aspects of the work in ensuring that questions related to the accuracy or integrity of any part of the work are appropriately investigated and resolved. AA and MB were involved in the analysis and interpretation of data for the work. JK drafted the work. AA and MB revised it critically for important intellectual content.

### Conflict of Interest

The authors declare that the research was conducted in the absence of any commercial or financial relationships that could be construed as a potential conflict of interest.
